# Deer antler stem cells are a novel type of cells that sustain full regeneration of a mammalian organ—deer antler

**DOI:** 10.1038/s41419-019-1686-y

**Published:** 2019-06-05

**Authors:** Datao Wang, Debbie Berg, Hengxing Ba, Hongmei Sun, Zhen Wang, Chunyi Li

**Affiliations:** 1 Institute of Special Wild Economic Animals and Plants, Chinese Academy of Agricultural Sciences, 4899 Juye Street, Changchun, 130112 China; 2 AgResearch Ltd, Ruakura Agricultural Centre, 10 Bisley Road, Hamilton, 3214 New Zealand; 3 Changchun Sci-Tech University, Changchun, 130600 China

**Keywords:** Mesenchymal stem cells, Stem-cell differentiation

## Abstract

Deer antlers are extraordinary mammalian organs that can fully regenerate annually. Antler renewal is a stem cell-based epimorphic process and antler stem (AS) cells can initiate de novo generation of antlers in postnatal mammals. However, although being called stem cells, the AS cells have not been characterized at molecular level based on the stem cell criteria. Comprehensive characterization of the AS cells would undoubtedly help to decipher the mechanism underlying the full regeneration of deer antlers, the only case of stem cell-based epimorphic regeneration in mammals. In the present study, three types of AS cells (antlerogenic periosteal cells APCs, for initial pedicle and first antler formation; pedicle periosteal cells PPC, for annual antler regeneration; and reserve mesenchyme cells RMCs, for rapid antler growth), were isolated for comprehensive molecular characterization. A horn-growth-related gene, RXFP2, was found to be expressed only in AS cells lineages but not in the facial periosteal cells (FPCs, locates geographically in the vicinity of the APCs or PPCs), suggesting the RXFP2 might be a specific marker for the AS cell lineage in deer. Our results demonstrated that AS cells expressed classic MSC markers including surface markers CD73, CD90, CD105 and Stro-1. They also expressed some of the markers including Tert, Nestin, S100A4, nucleostemin and C-Myc, suggesting that they have some attributes of the ESCs. Microinjection of male APC into deer blastocysts resulted in one female foetus (110 days gestation) recovered with obvious pedicle primordia with both male and female genotype detected in the ovary. In conclusion, the AS cells should be defined as MSCs but with partial attributes of ESCs.

## Introduction

Discovery of stem cells has revolutionized regenerative medicine and brought new hope for some of the diseases that are currently incurable^1,2^. Stem cells have been broadly classified by their origin into two types: embryonic and adult stem cells^3^. The former is from the inner cell mass of blastocysts and the latter retrieved from multiple tissue types (e.g. bone marrow, fat and Wharton’s jelly, etc.). Stem cells are extraordinary in that they are capable of self-renewal and can differentiate into multiple cell types, particularly embryonic stem cells (ESCs)^4^. Even though, it is still unbelievable if there exists a single type and limited number of residential cells that have stem cell attributes and can initiate de novo generation of appendages/organs in postnatal mammals; their progeny can sustain annually full regeneration of the appendages; and the derivative cells from the progeny can drive elongation of the appendage at an astonishing speed (up to 2 cm/day)^5^. The cells in this lineage are antler stem (AS) cells.

Deer antlers are male secondary sexual appendages that can fully regenerate once lost^6,7^. Antlers regenerate in yearly cycles: in spring, old ossified antlers are cast and nascent antlers start to grow from the permanent bony protuberances, known as pedicles; in summer, antlers rapidly elongate (up to 2 cm per day)^5^; antler growth slows down in late summer/early autumn and the antler becomes totally calcified in late autumn; in winter, fully calcified antlers are firmly attached to their living pedicles until the following spring, when they are cast again to trigger a new round of antler regeneration^8,9^. Deer antlers offer a unique opportunity to learn how nature has achieved full mammalian organ regeneration.

It is known that the antler growth centre is located in its tip^10,11^. Rapid antler growth is mainly achieved through the proliferation of cells resident in the reserve mesenchyme (RM), the outermost layer of the proliferation zone^12^. Therefore, the RM cells (RMCs) must have huge proliferation potential to sustain such a formidable growth rate. Morphological and histological studies have shown that the growth centre of regenerating antlers is initially formed from proliferation and differentiation of the pedicle (permanent bony protuberance) periosteal cells (PPCs)^13,14^. Tissue deletion^15^ and membrane insertion^16^ experiments further confirmed that annual antler regeneration fully relies on the presence of the pedicle periosteal (PP) tissue. Deer are not born with pedicles, which develop from deer frontal crests when deer approach puberty^17^. It is known that pedicle and first antler are initially formed from the frontal crest periosteum, known as antlerogenic periosteum (AP). Deletion of the AP abrogates pedicle and first antler formation and transplantation of the AP on the deer body can induce ectopic antler formation^18–20^.

The term “Antler stem (AS) cells” has been proposed to define the cells from the AP and PP^13,21^, and cells from the AP^22^, PP^23,24^ and RM^25,26^ have been isolated, cultured and partially characterized by several laboratories. Li et al.^6^ reported that Oct4, Sox2 and Nanog, known as core genes for pluripotency, were present in both the AP cells (APCs) and PPCs, and these cells could be induced in vitro to differentiate into adipocytes, chondrocytes, osteocytes and neuronal-like cells. Rolf et al.^23^ isolated Stro-1^+^ cells from the PP and RM, and defined these cells as a type of mesenchymal stem cells (MSCs). Seo et al.^25^ cultured antler-derived multipotent cells from the antler tips (roughly equivalent to the RMCs), and found that majority of them expressed CD105 (79%) and Oct4 (70%). These studies have shown convincingly that cells resident in the AP, PP and RM tissues possess stem cell attributes; therefore, antler regeneration has been deemed a stem cell-based epimorphic process^6^. Nevertheless, a comprehensive molecular characterization of AS cells is required to advance our understanding of this stem cell-based epimorphic regeneration.

In the present study, the APCs, PPCs and RMCs were all defined as AS cells. The AS cells were isolated from their respective tissues and subsequently cultured. As specific markers for the identification of AS cells are currently lacking, a combination of markers was used for the isolation and characterization of these AS cells. Expression status of the selected stem cell markers (both surface and intra-cellular) in the AS cells were investigated using both molecular characterization and in vitro differentiation. In vivo multipotency determination using APCs microinjected into expanded blastocysts and tissue analysis of the resulting fetus were conducted. Molecular characterization, in vitro differentiation and in vivo multipotency determination of the AS cells in the present study would help our understanding of the mechanism underlying antler regeneration, the only stem cell-based full organ regeneration in mammals.

## Materials and methods

### Tissue collection

All the tissue samples were collected following the methods reported by Li and Suttie^27^. The AP and the PP tissues were obtained from three 8-month-old and three 3-year-old male sika deer immediately after slaughter. The facial periosteum (FP, as a control tissue) was collected immediately after sampling of the AP or the PP. The RM was sampled from the growing antler tips 4 weeks after casting of the previous hard antler buttons. Location of tissue sampling is shown in Supplementary Fig. S1. Bone marrow was aspirated from the leg bones of six adult male sika deer immediately after slaughter. Three biological replicates for each tissue type were collected in this study. All tissue collections in this study were approved by CAAS Animal Ethics Committee (CAAS2016025).

### Cell culture

Cell culture was carried out using the methods reported by Li et al.^22^. Briefly, the sampled tissues were cut into small pieces and digested in Dulbecco’s Modified Eagle’s Medium (DMEM) containing collagenase (150 units/ml). After removing collagenase, digested complexes were cultured in DMEM medium containing 10% fetal bovine serum, 100 mg/ml of streptomycin and 100 units/ml of penicillin (Gibco, Grand Island, USA). AS cells were then frozen in a medium containing 10% DMSO and 90% serum when reaching sub-confluence. Cells for the experiment were less than passage 4. Bone marrow mesenchymal stem cells (BMSCs) were isolated using the deer lymphocyte separation medium (Haoyang, Tianjian, China). Released cells were placed into culture, and non-adherent cells were removed 48 h later. Lymphocytes were cultured in Roswell Park Memorial Institute 1640 (RPMI-1640) medium supplemented with 10% thermally inactivated serum, 100 mg/ml of streptomycin and 100 units/ml of penicillin. All cells were cultured in a humidified atmosphere with 5% CO_2_ at 37 ^o^C respectively.

### Colony formation assay

To evaluate the potential of self-renewal of the AS cells, the colony formation assay was carried out using a reported methodology^28^. Cells were cultured to 70–80% confluence and collected using trypsin; 200 cells were seeded into each well of 6-well plates and each treatment was performed in triplicate. Cells were maintained in the conditioned medium (collected from the same type as previously cultured cells) and medium was refreshed every 3 days; after 14 days of culture the cell colonies that had formed were washed with phosphate buffered saline (PBS), fixed in 4% paraformaldehyde for 30 min and stained with 0.5% crystal violet dye for 5 min. Each well was gently washed under tap water to remove any excess stain and then air dried for viewing. Photographs of the stained colonies were taken, and colony forming efficiency was assessed by the number of colonies from 100 cells.

### RNA-seq

Total RNA was extracted from the PP tissue using a Trizol reagent (Invitrogen Inc., Camarillo, CA) according to the manufacturer’s procedure. Three RNA-seq libraries of each samples were constructed using a TruSeq RNA sample preparation kit (Illumina Inc., San Diego, CA) according to the manufacturer’s instruction. The tagged cDNA libraries were loaded onto the channels of an Illumina HiSeq 2000 instrument and sequenced for 90 bp paired-end at Beijing Genomics Institute, Shenzhen, China. The reads that contained the sequencing adaptor and unknown bases (>2% “N”s per read) and the low-quality reads (>20% bases smaller than Q20 per read) were discarded. Then, Trinity package^29^ was run on the two clean read datasets to carry out a de novo assembly using the following parameters: the fixed default k-mer size of 25, k-mers minimum count of 4, minimum Contig length of 200, paired fragment length of 250 and other default settings. The following parameters were used to ensure high quality of the assembly: a minimum of 95% identity, a minimum of 40 overlapping bases and a maximum of 20 unmatched over hanging bases at sequence ends. BLASTx matches were performed between all unigenes and publicly-available protein databases, including NCBI non-redundant protein (Nr) database (http://www.ncbi.nlm.nih.gov), Swiss-Prot protein database (http://www.expasy.ch/sprot) and the Kyoto Encyclopedia of Genes and Genomes (KEGG) pathway database (http://www.genome.jp/kegg). The top BLASTx hit against Nr database were, then, annotated using Blast2GO. Stem cell markers were also retrieved and allocated into two categories, embryonic or adult stem cells, based on the criteria set by Pazhanisamy^30^.

### RT-PCR

Total RNA was extracted from the cultured cells of each AS cell type using total RNA Purelink kit (Bioteke, Beijing, China) following the manufacturer’s protocol. First-strand cDNA was synthesized from 1 μg of total RNA (DNase-treated) using a Primescript RT-PCR kit (Takara, Dalian, China). PCR amplifications of cDNA were performed in 25 μl solution using rTaq (Takara). PCR reaction conditions were set for 2 min at 94 °C, followed by 30 amplification cycles (94 °C for 30 s, 30 s at specific primer annealing temperature and 72 °C for 30 s). The sizes of the PCR products were estimated using 100-bp DNA marker on 1% agarose gel electrophoresis. The purified PCR products were directly sequenced by Sangon (Shanghai, China). All primers used in this study are listed in Supplementary Table 1.

### Western blotting

Total proteins were extracted from the cultured AS cells, BMSCs and FP cells respectively using RAPI lysis buffer (Beyotime, Jiangsu, China). Proteins from each cell extract were separated by using 12% SDS-PAGE gel and transferred to PVDF membranes (Amersham Biosciences, Piscataway, USA). The membranes were blocked with 5% (w/v) skim milk and incubated with each of primary antibodies (diluted according to manufacturer’s protocol) for 2 h at RT. Following washing with TBST for three times, the membranes were incubated with secondary antibody conjugated with HRP (1:3000) for 1 h at RT. Generated bands were visualized with an ECL detection reagents (Thermo Scientific, Rockford, USA), which were applied to the autoradiograph films.

### Immunofluorescent staining

Immunofluorescent staining was carried out as described elsewhere^31^. Briefly, cells were seeded at a density of 10,000/cm^2^ in 24-well plates with sterile cover slips. After 24 h of incubation, cover slips with adhered cells were rinsed with PBS and fixed in 4% formaldehyde for 30 min. After washing with PBS for three times, cells were incubated in blocking solution (5% BSA in PBS) for 1 h. For intracellular proteins, cells were permeabilized with blocking solution containing 1% TritonX-100. Cells were incubated with primary antibody for 1 h at RT, and isotype-matched rabbit or mouse IgG/IgM served as the negative controls. Cells were washed three times in PBS to remove excess primary antibody followed by incubation with the secondary antibody (ab150077, 1:500) conjugated with fluorescein for 1 h at RT in the dark. The nuclei of cells were counterstained with DAPI for 5 min at RT after removing excess secondary antibody. Cover slips were mounted onto glass slides with anti-fade reagent and examined under a fluorescent microscope. All antibodies used in this study are listed in Supplementary Table 2.

### Flow cytometry analysis

Surface markers for each type of AS cells were analyzed using flow cytometry as described elsewhere^32^. Briefly, cultured cells were incubated with 5% BSA to block non-specific staining and then incubated with primary antibody for 1 h at RT. Isotype-matched rabbit or mouse IgG served as the negative controls. After washing with cold PBS, cells were stained with secondary antibody conjugated with fluorescein for another 1 h at RT in the dark. Flow cytometry analysis was performed using FACSCalibur (BD biosciences), and a minimum of 30,000 events was collected for each sample. The results were analyzed using Cellquest software (BD biosciences). Histogram markers (M1) were placed according to the negative controls, and percent of positive cells were calculated using histogram statistics.

### Immunosuppression

The immunosuppression effects of the AS cells were tested using lymphocytes and were analyzed following a published method^33^. Briefly, 4 × 10^4^ cells were seeded to each well of 24-well plates. Peripheral blood mononuclear cells (PBMCs) were isolated from fresh samples using Deer Lymphocyte Separation Solution (Tbdscience, Tianjin, China). PBMCs were diluted to 10^7^ cells/ml in PBS containing 0.1% BSA and stained with 5 mM CFSE (Invitrogen). Labeled PBMCs were co-cultured with each type of AS cells at a ratio 1:10 (ASC to PBMC), in the presence of concanavalin A (Con A; 1 μg/ml). After 4 days of incubation, PBMCs were collected and attenuation of CFSE fluorescence intensity was assessed using flow cytometry.

### Multipotency

For osteoblast differentiation, each cell type was cultured in 6-well plates up to 70–80% confluence and maintained in the osteoblast differentiation medium (DMEM containing 10 nM dexamethasone, 100 mM L-ascorbic acid, 10 mM β-glycerophosphate I, and 5% FBS) for 21 days, with the medium changed every 3 days. To detect calcium deposition, cells were stained with 2% Alizarin Red S.

For adipocyte differentiation, each cell type was cultured up to 80–90% confluence and then shifted to adipogenic differentiation medium (DMEM containing 1 mM dexamethasone, 60 mM indomethacin, 500 mM 3-isobutyl-1-metyl-xanthine, and 10 mg/ml insulin) and cultured for a further 14 days. To detect accumulated lipids, cells were fixed in 4% formaldehyde, incubated in 60% isopropanol and stained with 0.5% Oil Red O.

For chondrogenic differentiation, each cell type was cultured in a micromass culture as the previously described^34^. Briefly, 2 × 10^5^ cells were spun down to make a pellet in a 15-ml conical tube, and the pellet was cultured in chondrogenic medium (DMEM containing 10 ng/ml recombinant human TGFβ (Sigma), 0.1 mM DEX, 50 mg/ml l-ascorbic acid 2-phosphate, 40 mg/ml l-proline (Sigma), and 1% ITS) for 14 days. The chondrogenic medium was replaced every 3 days. After 14 days culture, chondrogenic medium was replaced with hypertrophic induction medium (DMEM containing 50 ng/ml thyroxine (Sigma), 1 nM DEX, 20 mM β-glycerophosphate, 50 mg/ml l-ascorbic acid 2-phosphate, 40 mg/ml l-proline, and 1% ITS) for 28 days. Thereafter, nodules formed from micromass culture were fixed in 4% formaldehyde, embedded in paraffin, and cut into 6-µm sections. For histological evaluation, the sections were stained with hematoxylin and eosin and counterstained with Alcian blue-PAS.

### Chimera production

The multipotent capacity of APCs was tested in vivo by their ability to participate in the formation of chimeric fetuses. Two different red deer AP cell lines were used: one female and one male. AP tissues were collected, cell lines established and cryopreserved as described by Li et al.^27^ and the male line used previously by Berg et al.^35^.

Ovaries from red deer were collected at the local abattoir and oocytes aspirated, matured in vitro and fertilized as per Berg and Asher^36^. The presumptive zygotes were placed into 20 μl drops eDSOF 18 h post insemination; at 32 h post insemination, the cleaved embryos were placed into new drops of eDSOF, leaving the other presumptive zygotes to develop in the original culture drops. The early cleaving embryos were separated to skew the sex ratio to male^37^; later cleaving embryos were considered to be skewed to female embryos. All embryos were changed to late Deer Synthetic Oviduct Fluid (LDSOF) on day 5. On day 7, grade 1 and 2 expanded blastocysts were selected for microinjection. The female AP cells were injected into the presumptive male expanded blastocysts and the male AP cells were injected into the presumptive female expanded blastocysts. APCs were thawed, washed to remove the cryoprotectant and resuspended in LDSOF at a concentration of 10,000 cell per ml. Cells were held in a 40 μl drop under oil at 38.5 °C under 5% CO_2_ in air until microinjection. AP cells (10–15) were microinjected into the blastocoel cavity of each expanded blastocyst (using a 15–20 μm diameter beveled pipette). After injection, blastocysts were allowed to re-expand for 1 hour in LDSOF before transfer (n = 15; 4 putative female, 11 putative male) to recipient female deer as singletons. Morula-stage embryos were disassociated and 6 to 10 cells were microinjected into blastocysts and served as a positive embryonic cell control (n = 2). Recipient red deer females were synchronized and microinjected and control embryos transferred on day 7 of the cycle; pregnancy diagnosis using ultrasonography^35^ was performed on day 35, 42 and the day before fetal recovery. Pregnant uteri were recovered at slaughter on gestational day 102–110, a time when the pedicles can be clearly seen on male deer fetus^38^. Fetuses were recovered from the uterus, neck girth, crown-rump lengths and visual examination of pedicles were recorded. The appearance of the fetus and organs were noted. Representative tissue samples were taken and frozen in liquid nitrogen.

The presence of the opposite sex DNA in the fetal tissues was used to verify chimaerism. DNA extraction of 150 mg of tissue was performed^39^. PCR amplification of tissues representing the three lineages was conducted using two deer sex primers, amelogenin gene product of Pfeiffer and Brenig^39^ and the SRY gene product 116 bp and control DNA BOVIRBP 180 bp^40^.

### Statistical analysis

Data were presented as the mean ± SEM from three or more independent experiments. Statistical analysis was performed with *t* test using SAS (Statistical Analysis System) version 9.0, and *p* values < 0.05 were considered to be significant.

## Results

### Cell morphology and colony forming efficiency

**Fig. 1:**
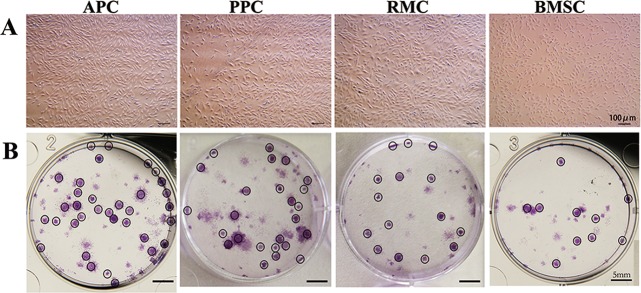
Morphological observation and colony formation of AS cells. **a** Cells from antler relative tissues were isolated and cultured as described in methodology section; their morphology was monitored under microscope (Passages ≤ 5); Bar = 100 μm. **b** Colonies formed after seeding 100 cells/well at day 14, and were stained with crystal violet dye; Bar = 5 mm; APC antlerogenic periosteal cell, PPC pedicle periosteal cell, RMC reserve mesenchymal cell, BMSC bone marrow stem cell

Three types of the AS cells and the BMSCs were isolated and cultured, morphologically they resembled fibroblasts (Fig. 1a). The ability of single cells to form colonies is a way to measure cell self-renewal, a key feature of stem cells. Single cells from each of the three types of the AS cells generated colonies on day 14, likewise did BMSCs (Fig. 1b). Colony forming efficiency of the APCs (15.8 ± 4.4%) and PPCs (13.5 ± 3.9%) were significantly higher (*p* < 0.05) than those of the BMSCs (5.1 ± 2.4%) and RMCs (6.5 ± 2.1%).

### Surface markers of stem cells

**Fig. 2:**
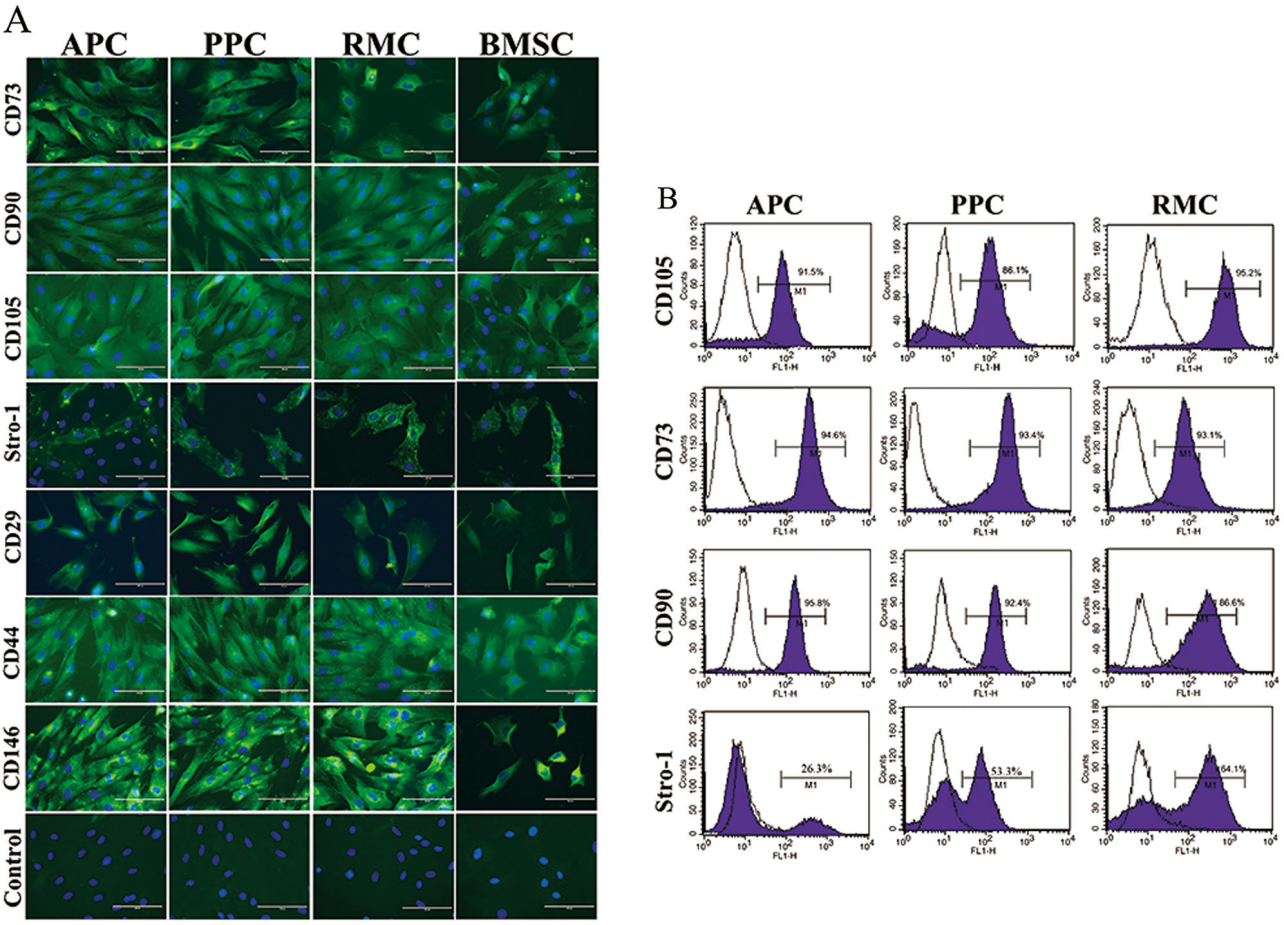
Expression of surface stem cell markers in AS cells. **a** Immunofluorescence staining of AS cells. Classical surface markers of stem cells (CD73, CD90, CD105, Stro-1, CD29, CD44, and CD146) were detected using immunofluorescence staining (Green). Cell nuclei were counterstained with DAPI (Blue). Scale bar = 100 µm. **b** Flow cytometry analysis of AS cells. Expressions of indicated antigen are shown in purple histograms in contrast to isotype controls (black histograms). Values showed positive expression patterns of the indicated antigen

CD9, an ESC marker, was defined as a surface marker of the AS cells in the previous studies^6,41^. We also detected its expression in all three types of AS cells in this study (Supplementary Fig. S2) using immunofluorescent staining and flow cytometry. CD73, CD90, CD105, CD29, CD44, CD146 and Stro-1 were reported as markers for MSCs^30^; all of these markers were found to be expressed in the three types of AS cells in this study through immunofluorescent staining (Fig. 2a).

The results from flow cytometry analysis showed that majority of the three types of AS cells were positive to CD73, CD90 and CD105 (Fig. 2b). The expression levels of Stro-1 were low in the three types of AS cells but there was evidence of a pattern with the order of expression being: APCs (26.3%) < PPCs (53.3%) < RMCs (61.5%). That is, the more differentiated the AS cells, the higher the expression level of Stro-1, indicating that along the antler differentiation ontogeny, the AS cells are changing in potency from nearly pluripotent (ESC-like) to more restricted multipotent (MSC-like). The expression status of classical stem cell markers in these AS cells is shown in Table 1 and Supplementary Fig. S3.

**Table 1:** Expression of stem cell markers in the AS cells

Symbol	ASC				PPC				RMC			
	RT-PCR	WB	IF	FCM	RT-PCR	WB	IF	FCM	RT-PCR	WB	IF	FCM
Oct4	N	N	—	—	N	N	—	—	N	N	—	—
Nanog	N	N	—	—	N	N	—	—	N	N	—	—
Sox2	N	N	—	—	N	N	—	—	N	N	—	—
CD73	Y	Y	Y	>90%	Y	Y	Y	>90%	Y	Y	Y	>90%
CD90	Y	Y	Y	>90%	Y	Y	Y	>90%	Y	Y	Y	>80%
CD105	Y	Y	Y	>90%	Y	Y	Y	>80%	Y	Y	Y	>90%
Stro-1	—	—	Y	25%	—	—	Y	50%	—	—	Y	60%
Nestin	—	Y	Y	—	—	Y	Y	—	—	Y	Y	—
CD9	—	Y	Y	>95%	—	Y	Y	>90%	—	Y	Y	>90%
CD29	—	Y	Y	—	—	Y	Y	—	—	Y	Y	—
CD44	Y	Y	Y	—	Y	Y	Y	—	Y	Y	Y	—
CD146	—	Y	Y	—	—	Y	Y	—	—	Y	Y	—

*RT-PCR* reverse transcription-polymerase chain reaction, *WB* western-blot, *IF* immunofluorescence, *FCM* flow cytometry, *N* undetected, *Y* detected, — untested

RXFP2, a gene that is known to control horn phenotype expression^42^, was found to be highly expressed in the three types of AS cells, while it was undetectable in the FPCs (Supplementary Fig. S4).

### Intra-cellular markers of stem cells

**Fig. 3:**
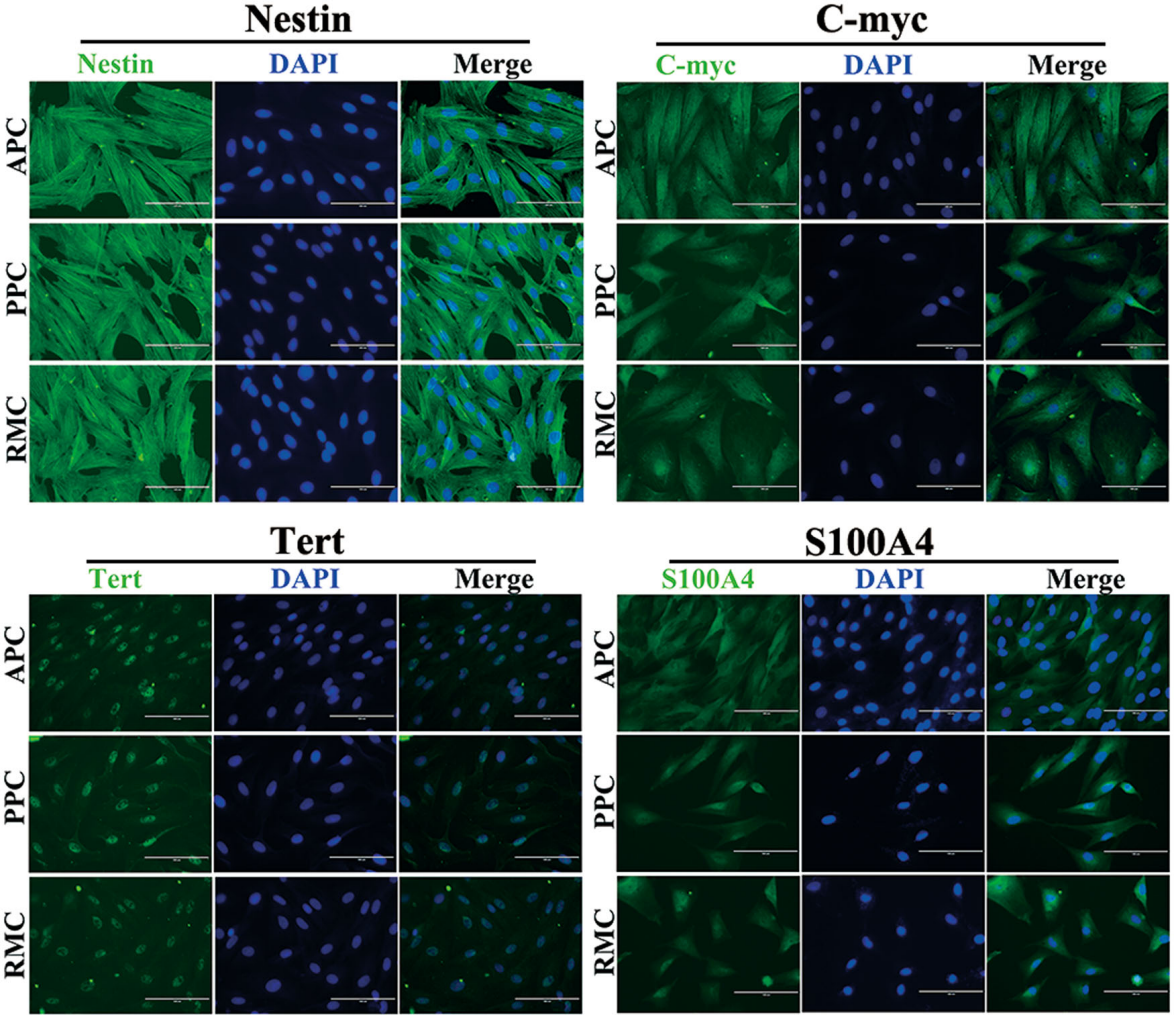
Immunofluorescence staining of intracellular markers in the AS cells. Nestin, C-myc, Tert and S100A4 (known as intracellular markers) were detected using immunofluorescence staining. Cell nuclei were counterstained with DAPI (Blue). Note that filamentous Nestin distributed in the whole cytoplasm and Tert enriched in cell nuclei. Scale bar = 100 µm

Three core ESC markers, Oct4, Sox2 and Nanog, that were previously reported to be expressed in the AS cells^6,23,25^, were not detected in this study using RT-PCR (Supplementary Fig. S5), Immunofluorescent staining demonstrated that all three types of the AS cells expressed high levels of filamentous Nestin in the cytoplasm, c-Myc and S100A4 in the cytoplasm, and Tert in the nucleus (Fig. 3).

### Transcriptome profiling

**Fig. 4:**
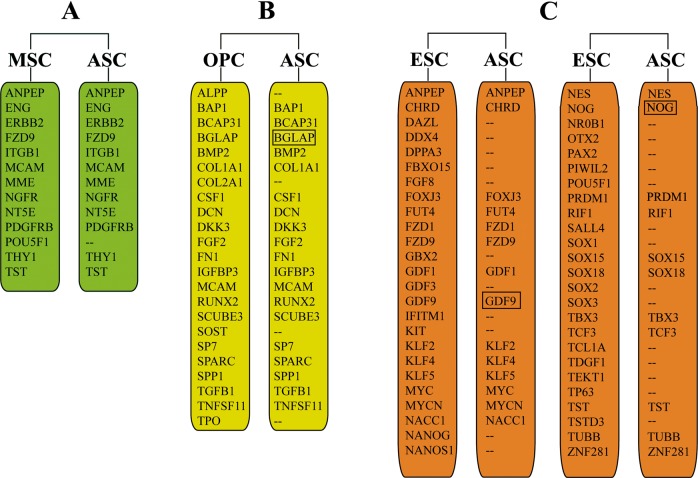
Expression of stem cell markers in AS cells (ASC). **a** Expression of mesenchymal stem cell (MSC) markers. **b** Expression of osteoprogenitor cells (OPC) markers. **c**: expression of embryonic stem cell (ESC) markers. The list of indicated stem cell markers were defined by Pazhanisamy (2013)^30^

The transcriptome of each type of AS cells was sequenced using Illumina HiSeq 2000 platform. Genes or sequences coding for transcriptional factors related to stem cells were further retrieved from the annotated sequence datasets. Based on a list of known stem cell markers^30^, 53 expressed genes from the present study were found to fall in the category of stem cells (Supplementary Tables 3a, b, c): 12 genes belong to MSCs (13 genes currently-known in total), 19 genes to osteoprogenitor cells (23 genes currently-known in total), and 25 genes to ESCs (50 genes currently-known in total) (Fig. 4).

### Multipotency in vitro

**Fig. 5:**
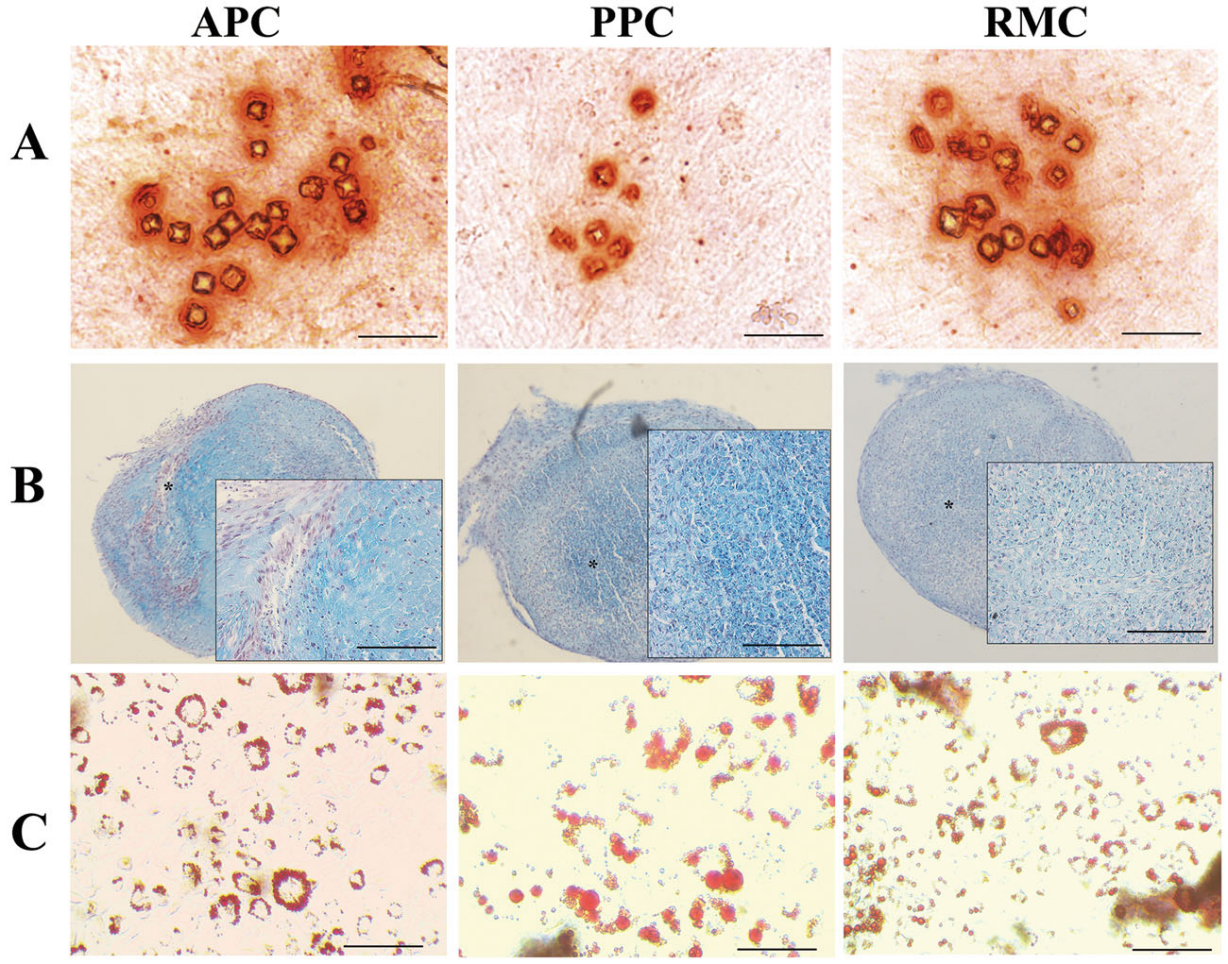
Multipotency of AS cells. **a** Osteogenic differentiation - Alizarin Red S and Von Kossa staining after 3 weeks of osteogenic induction; differentiated cells stained strongly with Alizarin Red S. **b** Chondrogenic differentiation - chondrogenic pellets were cut into 5-mm sections for Alcian blue-PAS staining; the stained tissue displayed a typical cartilaginous tissue phenotype. **c** Adipogenic differentiation - Oil Red O staining was conducted after 2 weeks of adipogenic induction; AS cells exhibited Oil Red-O positive lipid droplets

To investigate the multiple differentiation potentials of the AS cells in vitro, they were cultured in different types of media. When cultured in the osteogenic induction medium for 21 days, AS cells were strongly stained with Alizarin Red S (Fig. 5a); in the chondrogenic induction medium, a cell nodule was formed after a 28-day micromass culture and stained strongly with Alcian blue-PAS (Fig. 5b); and in the adipogenic induction medium for 14 days, the majority of the cells accumulated lipid droplets in their cytoplasm and stained positively with Oil Red O (Fig. 5c). These results demonstrate that AS cells can be readily induced to differentiate into alternative cell lineages and therefore can be classified as multipotent.

### Immunosuppression to the ConA-activated PBMCs

**Fig. 6:**
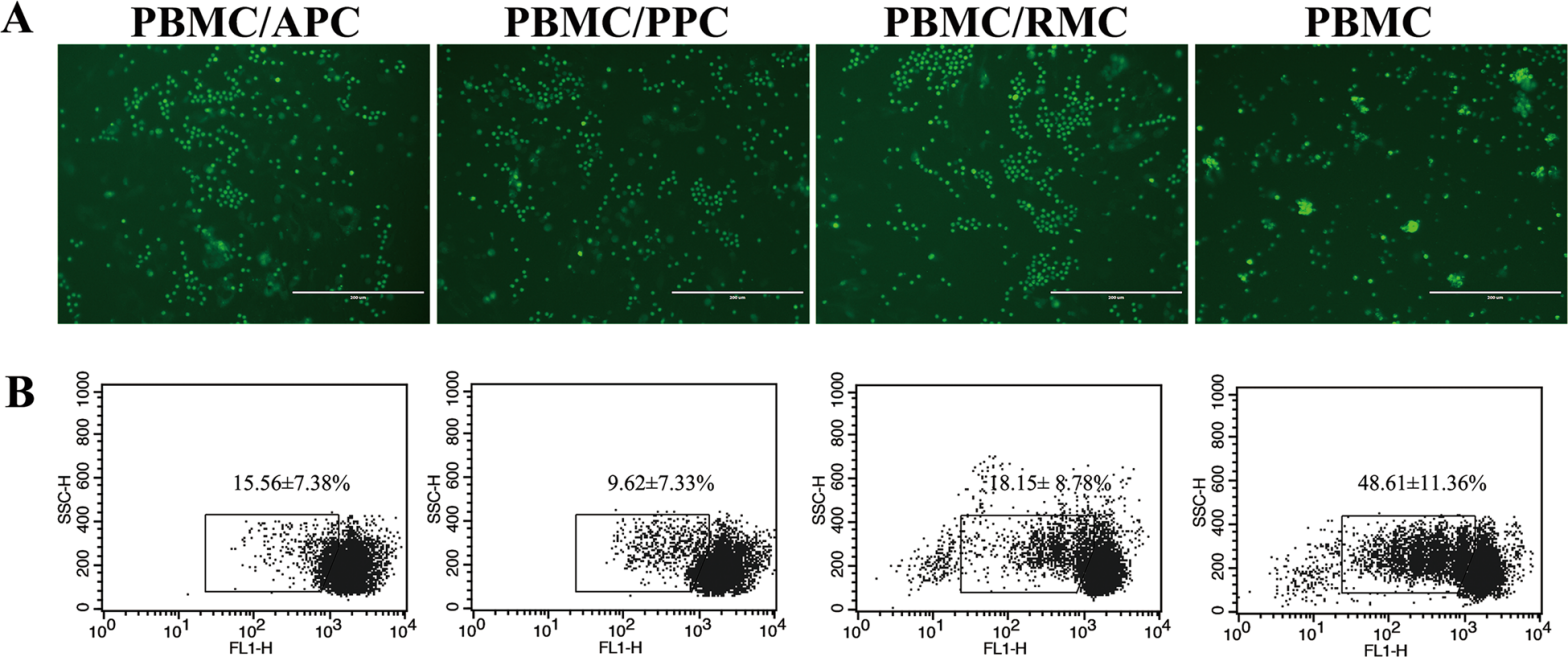
AS cells significantly suppressed the proliferation of active lymphocytes. **a** Lymphocytes were labelled with CFSE and co-cultured with AS cells at a ratio of 1:10 (ASC: lymphocyte) for 4 days and observed under fluorescence microscope; cell masses were proliferating lymphocytes; note that the co-cultured lymphocytes were almost devoid of cell masses. **b** Reduction of CFSE fluorescence intensity in PBMCs was analyzed by flow cytometry; data are expressed as the mean ± SD from three independent experiments

To test immunosuppressive activity of the AS cells, we examined the proliferative response of the ConA-activated PBMCs to the co-cultured AS cells. Compared to the singularly cultured ConA-activated PBMCs, each type of the co-cultured AS cells significantly suppressed proliferation of the ConA-activated PBMCs (*p* < 0.01), and proliferation rates of the PBMCs dropped significantly to 15.56 ± 7.38% (+APC), 9.62 ± 7.33% (+PPC) and 18.15 ± 8.78% (+RMC), respectively (Fig. 6).

### In vivo chimera production

**Fig. 7:**
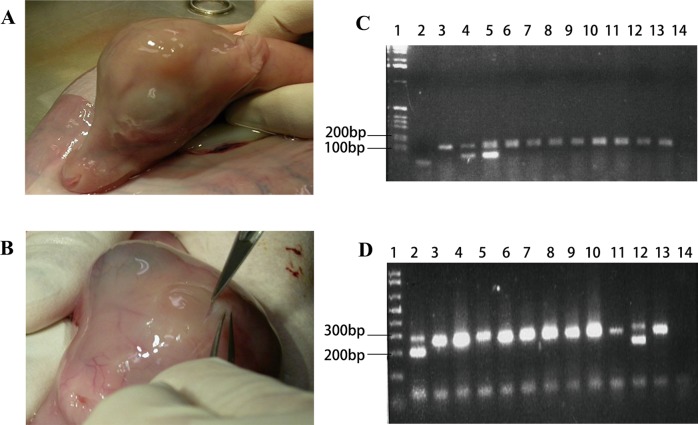
Production of chimera from APCs. **a** Male fetus derived from blastocyst injected with morula embryo cells; neck girth of 9.0 cm. **b** Female fetus derived from blastocyst injected with male AP cells. Note the prominent pedicle formation on the head; neck girth of 9.5 cm. **c** PCR amplification of SRY region of the Y chromosome (male 104 bp) and internal DNA positive control (194 bp) of various tissues from the female fetus with pedicles. (1) Ladder; (2) Water control; (3) Female control; (4) Male control; (5) Ovary (male DNA in the ovary); (6) Kidney; (7) Skin; (8) Pedicles; (9) Skin pedicles; (10) Heart; (11) Gut; (12) Brainstem; (13) Muscle; (14) Blood. **d** PCR amplification of amelogenin gene (male sequence deletion 200 bp) and female 300 bp of various tissues from the female fetus with pedicles. (1) Ladder; (2) Ovary (male DNA in the ovary); (3) Kidney; (4) Skin; (5) Pedicles; (6) Skin pedicles; (7) Heart; (8) Gut; (9) Brainstem; (10) Muscle; (11) Blood; (12) Male control; (13) Female control; (14) Water control

To investigate the multiple differentiation potential of the AS cells in vivo, the APCs were injected into the inner cell mass of deer blastocysts. Red deer (*Cervus elaphus*) embryo technology is well established and served as the model to assess the multipotency of deer APC cells. The pregnant rates at 35 days of gestation for the AP group were 43% (6/14), and at fetal recovery on day 102–110 was 36% (5/14). Five of the seven pregnancies resulted from APC microinjection and the remaining two were embryonic cell microinjections. Fetal morphometric measurements are presented in Supplementary Table 4. APCs from male deer were detected in ovary of one chimera (Fig. 7), and prominent pedicle primordia were observed on the head of one female fetus derived from an embryo injected with male APCs indicating chimerism (Fig. 7b). One AP presumptive chimeric female fetus exhibited both male and female genotype in the gonadal tissue while only the female genotype was detected in the other tissue tested (Fig. 7c, d). One of the two embryonic cell control transfers produced a fetus which was male and only male genotype was detected in all tissues tested.

## Discussion

To the best of our knowledge, this is the first comprehensive molecular characterization to the AS cell lineages (APCs, PPCs and RMCs). These results: (1) experimentally verified self-renewal capability (colony forming efficiency); (2) confirmed expression of almost all MSC markers but not the previously-identified three core ESC markers (Oct4, Nanog and Sox2), which made the AS cells more like the MSCs rather than ESCs as previously suggested; (3) found potent immunosuppressive ability; (4) produced a chimera after microinjection into a deer blastocyst, and (5) identified a possible specific surface marker gene for AS cells, namely RXFP2.

Although the AS cells reside in the periosteal tissues in postnatal animals, they were reported to express some key embryonic stem cell marker genes, such as Oct4, Nanog and Sox2^6,25^. Therefore, Li et al.^6^ proposed that the AS cells should be considered as an intermediate type of stem cells between the ESCs and MSCs. In contrast to these previous reports, we failed to detect expression of these key marker genes through transcriptome sequencing or RT-PCR in this study. In another study, we demonstrated that previously reported expression of the Nanog gene in the AS cells was actually a pseudogene^43^. In fact, the issue as to whether MSCs express ESC marker genes is still controversial. Some researchers reported that MSCs express Oct4 and Nanog^44,45^ but others reported that human MSCs expressed Nanog but not Oct-4 and Sox-2^46^. Therefore, whether these key ESC marker genes are expressed in the AS cells still remains.

In this study, we found that the AS cells expressed the classical MSC marker genes including CD73, CD90, CD105 and Stro-1, and 12 out of 13 MSC-related genes were detected in the transcriptome of AS cells. Therefore, if the AS cells are an intermediate type, as previously suggested by Li et al.^6^, they must be more inclined to the MSC side, although they should be considered a special type of MSC, as they are capable of the initiation/ generation of an entire organ and full regeneration of head appendages in postnatal mammals.

The capacity for extensive self-renewal and the capability to differentiate into diverse cell lineages are distinctive features of stem cell populations. The origin of the PP is the AP – where the PP differentiates from about 5.0 million APCs. Given that the AP provides around 3.3 million cells to each round of antler regeneration (up to 15 kg tissue mass/year) for the deer’s entire life^9^, this means that the AS cells must possess considerable capacity for self-renewal. However, this study is the first to demonstrate convincingly that singularly cultured AS cells could form large colonies in vitro which is a well-accepted criterion for testing the capacity of cell self-renewal.

Previous studies reported that the APCs and PPCs are multipotent in that they can be induced to differentiate into multiple cell lineages, including chondrocytes, osteoblasts, adipocytes, myotubes and neuronal-like cells^23,25^. However, Daley et al.^47^ reported that antlerogenic progenitor cells (roughly equivalent to RMCs in this study) in white-tailed deer are more lineage-restricted osteo/chondrogenic progenitors, with little adipogenic capacity. These findings are in contrast to those of Rolf et al.^23^ in fallow deer (*Dama dama*) and Seo et al.^25^ in sika deer (*Cervus nippon*), wherein the RMCs were readily induced to differentiate into adipocytes. In the present study, all three types of the AS cells were induced to differentiate in vitro into chondrocytes, osteocytes and adipocytes. However, we sampled RMCs at the early phase of rapid antler growth but in contrast, Daley et al.^47^ collected RMCs from the late phase of rapid antler growth; that is, the difference between the two studies may be a function of the timing of tissue sampling. Based on the results of our experiments, we can conclude confidently that the cells from the AS cell lineage are multipotent and that AS cells satisfy the criteria established for stem cells and are therefore, bona fide stem cells.

Nestin, best known as a marker of neural stem cells, is an intermediate filament protein and is involved in the radial growth of axons^48^. For example, it is reported that human hair follicle stem cells express high levels of Nestin and that these cells can be readily induced to differentiate into neurons and smooth muscle cells in vitro^49^. APCs have also reported to be able to differentiate into muscle precursor cells and neuronal-like cells^6^. Therefore, the AS cells may have the potential to differentiate into nerve cells in vivo given that antlers are richly innervated and the exons of these nerves elongate at phenomenal speed (up to 2 cm/day).

It is well known that c-Myc, one of the four Yamanaka factors, plays an essential role in the generation of induced pluripotent stem cells (iPS cells)^50^. We found that AS cells expressed high levels of c-Myc, indicating its possible role in maintaining pluripotency of the AS cells. This may be necessary as growing antlers comprise multiple cell types, into which the AS cells may be able to differentiate. Another important factor may be Tert, which is considered is indispensable for the stability of stem cells and cancer cells through its role in maintaining telomere length^51^. In the present study, we found that Tert was highly expressed in the AS cells; this is not surprising, given that importance of maintaining telomere length for the integrity of cell division, as around 3.3 million PPCs can form 10 kg or so antler tissue mass within 60 days. A calcium-binding protein, S100A4, is reported to be highly expressed in many embryonic and cancer tissues^52^ and is a strong factor for promoting cell proliferation and tumor growth^53^. In the present study, we found that the S100A4 gene was highly expressed in the AS cells, suggesting that it may play a significant role in the rapid growth of antlers.

MSCs are known to have potent immuno-modulatory effects, such as suppression of lymphocyte proliferation both in vitro and in vivo^54^. In our study, the AS cells suppressed proliferation of ConA-activated lymphocytes significantly (Fig. 6), providing further evidence for the claim that AS cells meet the criteria for stem cells. Moreover, understanding the interactions between the AS cells and immune cells may hold one key to the mechanism underlying antler regeneration.

Both in vitro multipotency and the RNA-seq results suggest that the AS cells have characteristics of both MSC and ESC. We then asked the question whether AS cell would contribute to multiple lineages in vivo after microinjection into a host deer blastocyst, to determine whether the AS cells were more ES-like than MCS-like. We used the sex genotype to determine chimerism in deer fetuses at 102 to 110 days gestational ages. Antler pedicles along with an increase in neck girth are apparent in the male deer fetus from 60 to 110 days of gestation and accompany a surge in testosterone secreted by the fetal gonad^55^. We used these secondary sex characteristics to screen for potential female/male chimeras, and one female fetus with an obvious pedicle had both male and female genotypes in the ovary, but only the female genotype was detected in the pedicle tissue. We speculate this may be in response to testosterone produced by male tissue in the gonad, since testosterone was detected in the deer fetal gonadal tissue^55^. Further chimeric experiments are required using a transgene marker in the AS cells for further histological evidence of lineage development potential in this type of cells.

The relaxin/insulin-like family peptide receptor 2 (RXFP2), also known as LTG8, is a G-protein coupled receptor for glycoprotein hormones^56^. RXFP2 was initially identified by Johnston et al.^42^ as a singular factor that is associated with the sheep horn phenotype. The finding that an 1833-bp genomic insertion in the 3′-UTR region of RXFP2 causes the sheep hornless phenotype provides convincing evidence that the RXPF2 gene controls horn phenotype^57^. Interestingly, we found that the RXFP2 gene was expressed at relatively high levels in the AS cell lineage in this study, but not in the control cell FPCs. As both sheep horns and deer antlers are cephalic appendages, we infer that the RXFP2 gene may also be involved in antler development. Irrespective of its possible role in antler formation, RXFP2 can be used as a specific marker for the isolation of AS cells and effectively used to distinguish them from FPCs. This is useful as the FPCs were located in the vicinity of the APCs and PPCs, and share many common features with and could not be readily distinguished from AS cells at the molecular level in previous studies. Therefore, the RXFP2 gene is thus far the only marker that can be used to distinguish the AS cells from the FPCs.

In the present study, we have conducted comprehensive characterization of the three distinct cell types of AS cells and found that they exhibit almost all the features defined for mesenchymal stem cells. Besides, the AS cells had attributes that are shared in part with ESC; therefore, the AS cells may be best defined as a special type of MSC that possess some ESC features. In any case, comprehensively characterizing and defining the AS cells should provide valuable insights into mammalian organ regeneration and the rapidly developing field of human regenerative medicine.

## Supplementary information

Suppl Figure S1

Supplementary Table 1

Supplementary Table 2

Suppl Figure S2

Suppl Figure S3

Suppl Figure S4

Suppl Figure S5

Supplementary Table 3a

Supplementary Table 3b

Supplementary Table 3c

Supplemental Table 4

Supplementary figure legends
