# Supplementary figures and images for: Deer antler stem cells are a novel type of cells that sustain full regeneration of a mammalian organ—deer antler
Source: Cell Death Dis. 2019 Jun 5;10(6):443. doi: 10.1038/s41419-019-1686-y (PMC6549167; doi:10.1038/s41419-019-1686-y)

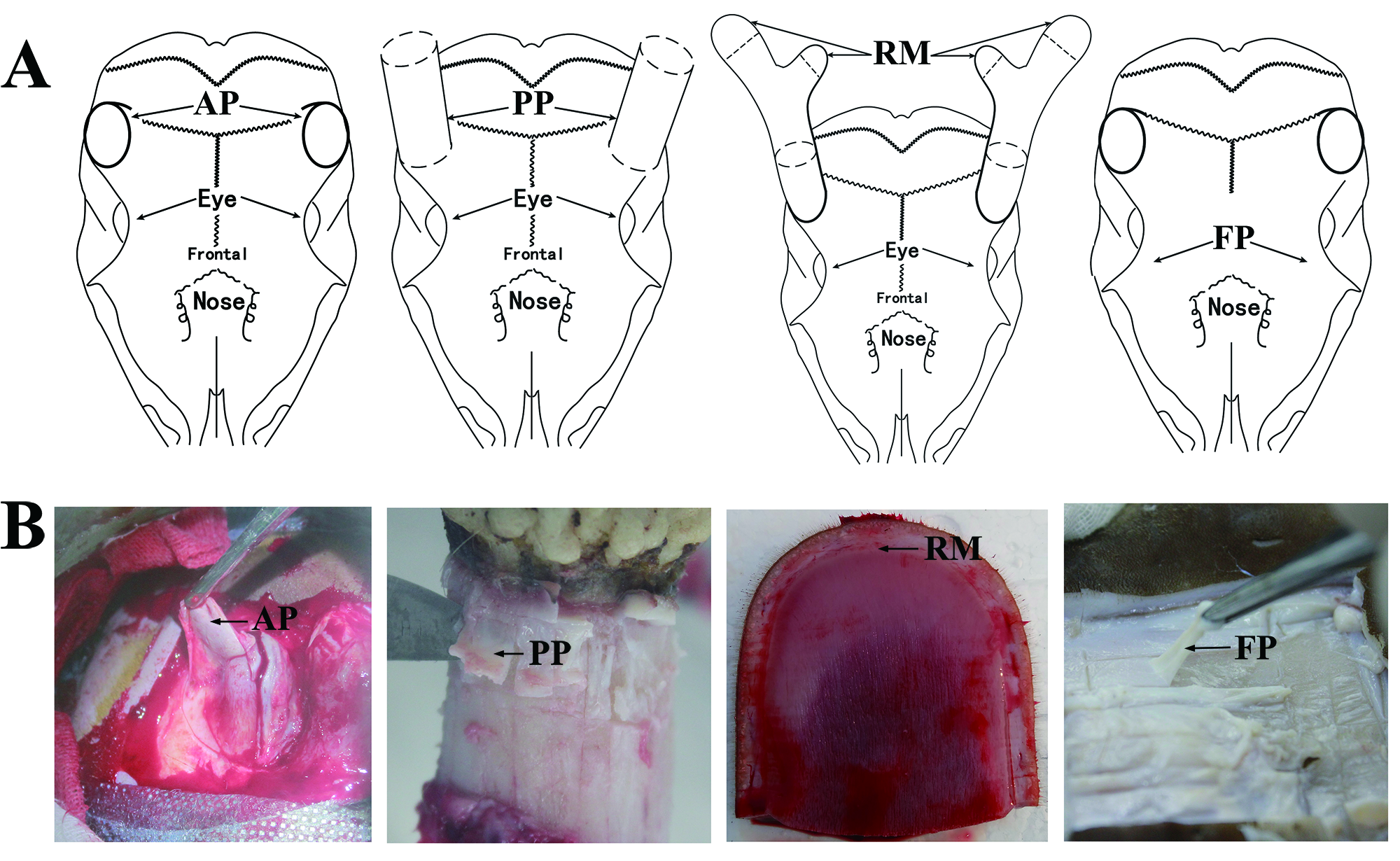

Supplement: Supplementary file 1 — Suppl Figure S1 [file 41419_2019_1686_MOESM1_ESM.tif]

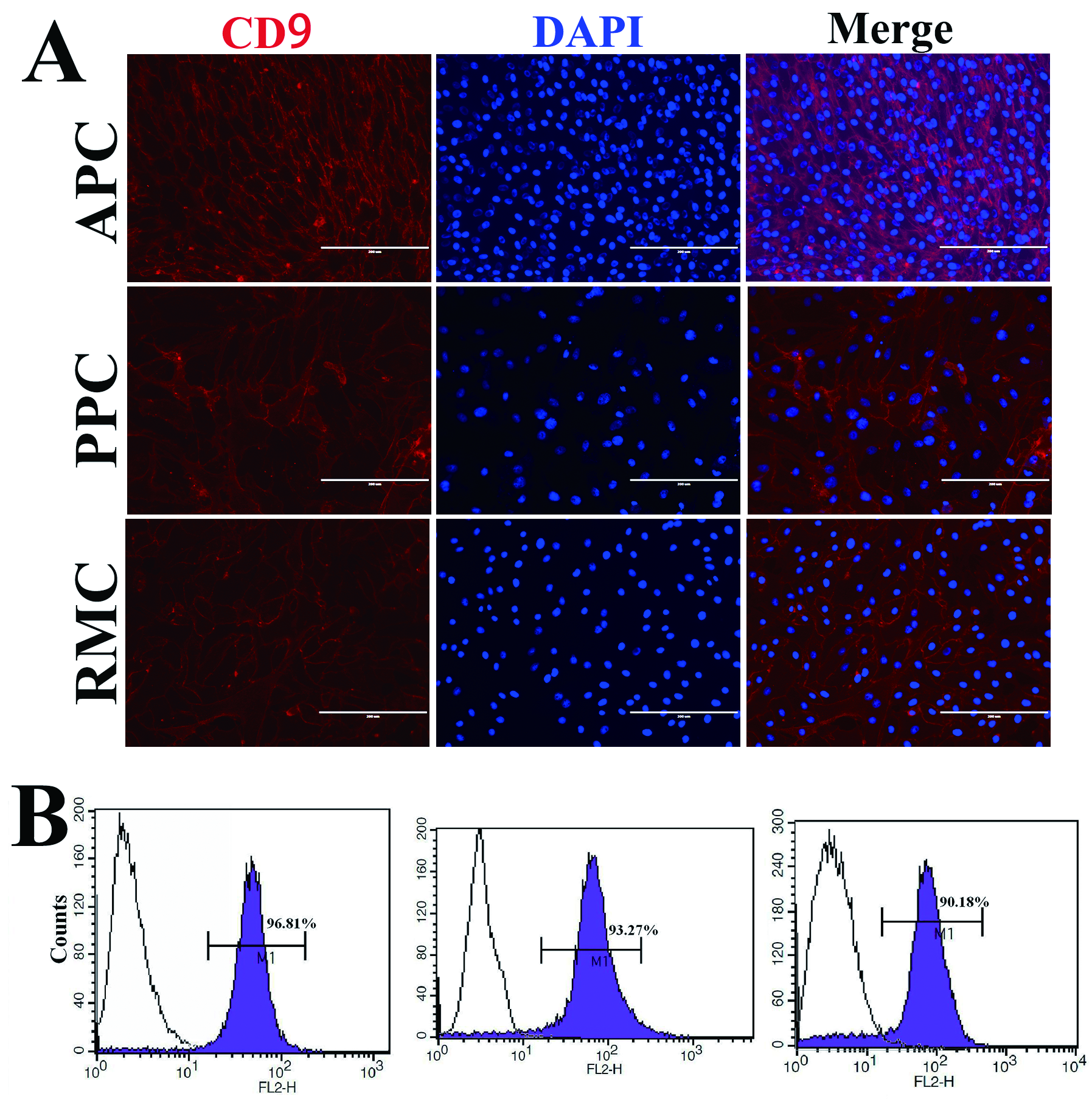

Supplement: Supplementary file 4 — Suppl Figure S2 [file 41419_2019_1686_MOESM4_ESM.tif]

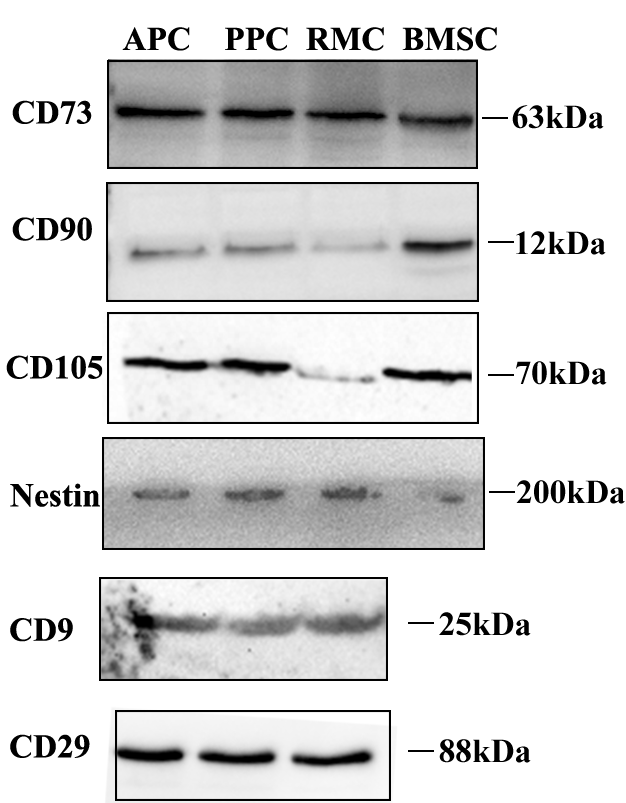

Supplement: Supplementary file 5 — Suppl Figure S3 [file 41419_2019_1686_MOESM5_ESM.tif]

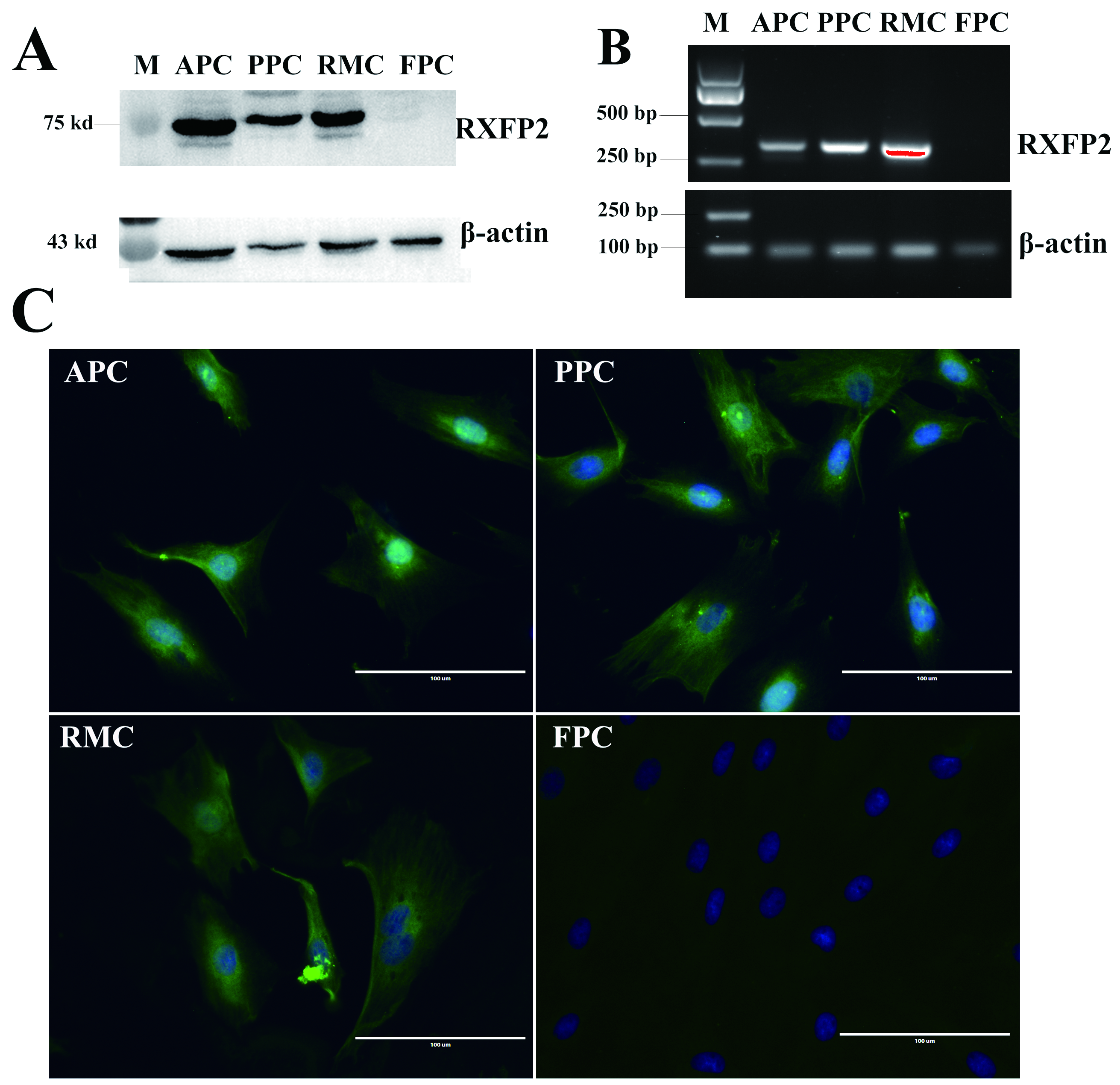

Supplement: Supplementary file 6 — Suppl Figure S4 [file 41419_2019_1686_MOESM6_ESM.tif]

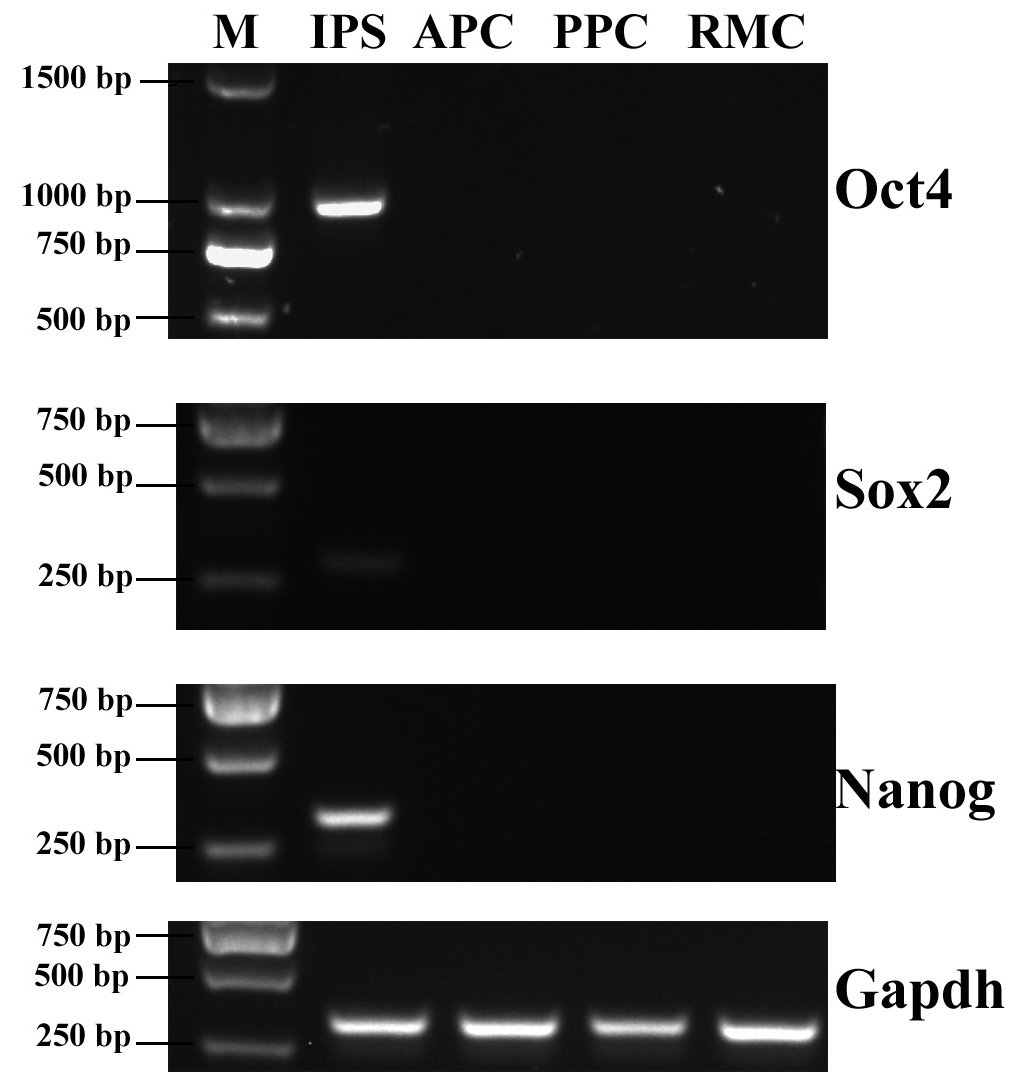

Supplement: Supplementary file 7 — Suppl Figure S5 [file 41419_2019_1686_MOESM7_ESM.tif]
