# Supplementary material for: Deer antler stem cells are a novel type of cells that sustain full regeneration of a mammalian organ—deer antler
Source: Cell Death Dis. 2019 Jun 5;10(6):443. doi: 10.1038/s41419-019-1686-y (PMC6549167; doi:10.1038/s41419-019-1686-y)
Supplement: Supplementary file 2 — Supplementary Table 1 [file 41419_2019_1686_MOESM2_ESM.docx]

Supplementary Table1 Primers used in this study

| Gene | Forward primer (5'-3') | Reverse primer (5'-3') | Product size (bp) |
| --- | --- | --- | --- |
| CD73 | GGTCAAAGGTGCCTCCAATG | CAATCCCATTCTTCTCAACAGC | 355 |
| CD90 | TCAGCCTGACAGCCTGCCTG | CTTATGCCCCCACACCTGAC | 331 |
| CD105 | TTCTGCCGCTTGGAAGGTGT | CTTGCCATCGCTGGAGCACTT | 480 |
| CD44 | CCTCCAGATGATGAGATGAGCA | CAAGATGATCAGCCATTCTGGA | 301 |
| Oct4a | GTGGAGGAAGCTGACAACAA | AGCCTGGGGTACCAAAATG | 352 |
| Oct4b | GATGTGGGGCTCACCCTGG | TTCGGGCACTGCAGGAACA | 265 |
| Sox2a | ACATGATGGAGACGGAGCTGAAG | GCTGATCTCCGAGTTGTGCATCT | 218 |
| Sox2b | CCAAGACGCTCATGAAGAAGG | CGTTCATGTAGGTCTGCGAGC | 309 |
| Nanog | CACCCTCGACACGGACACT | CTGCTTGTAGCTGAGGTTCAA | 281 |
| RXFP2 | CCTTGAACCCAATCCTCT | TCATCTTCCAAACGCTGT | 276 |
| Actin | CCGAGACATCAAGGAGAAGCTG | GTAGTTTCGTGAATGCCGCA | 206 |
