# Supplementary material for: Deer antler stem cells are a novel type of cells that sustain full regeneration of a mammalian organ—deer antler
Source: Cell Death Dis. 2019 Jun 5;10(6):443. doi: 10.1038/s41419-019-1686-y (PMC6549167; doi:10.1038/s41419-019-1686-y)
Supplement: Supplementary file 3 — Supplementary Table 2 [file 41419_2019_1686_MOESM3_ESM.docx]

Supplementary Table 2 Antibodies used in this study

| Terms | Manufacturer and product code | Application |
| --- | --- | --- |
| Mouse anti-CD9 | LSBio;LS-C46004 | WB^1^, IF^2^, Flow^3^ |
| Rabbit anti-CD29 | Biotech; ABIN2957829 | WB, IF, Flow |
| Rabbit anti-CD44 | Proteintech; 15675-1 | IF |
| Rabbit anti-CD73 | Santa; sc-25603 | WB, IF, Flow |
| Rabbit anti-CD90 | Bioss; bs-0778R | WB, IF, Flow |
| Mouse anti-CD105 | Elabscience; ESH135 | WB, IF, Flow |
| Rabbit anti-CD146 | Bioss; bs-1618R | IF |
| Mouse anti-Stro-1 | R&D; MAB1038 | IF, Flow |
| Rabbit anti-Nestin | Gene Tex; GTX39578 | WB, IF |
| Rabbit anti-C-myc | Elabscience; ENT0990 | IF |
| Rabbit anti-S100A4 | Abcam; ab27957 | IF |
| Rabbit anti-Tert | Elabscience; ENT4606 | IF |
| Rabbit anti-RXFP2 | Self-produced | WB, IF |
| Rabbit IgG-Isotype control | Abcam, ab172730 | IF, Flow |
| Mouse IgG-Isotype control | Abcam, ab37355 | IF, Flow |
| HRP-conjugated goat anti-mouse IgG | Beyotime; A0216 | WB |
| Goat anti-rabbit IgG H&L (Alexa Fluor 488) | Abcam; ab150077 | IF, Flow |
| Goat Anti-mouse IgG H&L (Alexa Fluor 488) | Abcam; 150113 | IF, Flow |
| Goat Anti-mouse IgM (Alexa Fluor 488) | Abcam; 150121 | IF, Flow |

^1^WB, Western-blot; ^2^IF, Immunofluorescence; ^3^Flow, Flow cytometry
