# Supplementary material for: Deer antler stem cells are a novel type of cells that sustain full regeneration of a mammalian organ—deer antler
Source: Cell Death Dis. 2019 Jun 5;10(6):443. doi: 10.1038/s41419-019-1686-y (PMC6549167; doi:10.1038/s41419-019-1686-y)
Supplement: Supplementary file 11 — Supplemental Table 4 [file 41419_2019_1686_MOESM11_ESM.docx]

Supplemental Table 4 Morphometric measurements of the 110 day red deer foetuses

| **Cell type & sex/fetal sex** | **Fetal weight (g)** | **Crown rump (cm)** | **Neck girth (cm)** | **Pedicles** |
| --- | --- | --- | --- | --- |
| F APS/male | 599 | 26.0 | 10.4 | Yes |
| F APC/male | 563 | 26.0 | 9.9 | Yes |
| M APC/female | 192 | 20.0 | 6.5 | No |
| M APC/female | 557 | 28.2 | 9.5 | Yes |
| M APC/male | 636 | 26.5 | 11.2 | Yes |
| Embryo/male | 453 | 26.7 | 9.0 | Yes |
| Embryo/male | 617 | 25.2 | 10.0 | Yes |

F APC-female APCs injected into blastocysts

M APC-male APCs injected into blastocyst

Embryo- morula embryo disaggregated and injected into blastocysts
