# Supplementary material for: Deer antler stem cells are a novel type of cells that sustain full regeneration of a mammalian organ—deer antler
Source: Cell Death Dis. 2019 Jun 5;10(6):443. doi: 10.1038/s41419-019-1686-y (PMC6549167; doi:10.1038/s41419-019-1686-y)
Supplement: Supplementary file 12 — Supplementary figure legends [file 41419_2019_1686_MOESM12_ESM.docx]

Supplementary Fig. S1 Sampling of antler related tissues. A: Schematic drawing of the location of antler related tissues on the deer head. B: Tissues were collected from deer. AP, antlerogenic periosteum; PP, pedicle periosteum; RM, reserve mesenchyme; FP, facial periosteum.

Supplementary Fig. S2 Expression of CD9 in AS cells. A: Immunofluorescence staining of cell surface marker CD9 (Red); cell nuclei were counterstained with DAPI (Blue). Bar=200 µm. B: Flow cytometry analysis - expressions of CD9 were shown in purple histograms, and black histograms were isotype controls. Values show positive expression patterns.

Supplementary Fig. S3 Expression of classical stem cell markers in AS cells, analysed using western blot.

Supplementary Fig. S4 Expression of RXFP2 at both mRNA and protein levels in the AS cells. A: Western blot analysis. Note that RXFP2 was solely expressed in the antler stem cell lineages. B: RT-PCR. Note that expression status of RXFP2 at transcription level was consistent to that at translational level, i.e. also in the antler stem cell lineage but not in FPCs; C: Immunofluorescent staining. Note that RXFP2 was specifically expressed in the cultured AS cells. Bar=100 μm.

Supplementary Fig. S5 Expression of embryonic stem cell makers in AS cells analyzed using RT-PCR.
